# Selective host autophagy is induced during the intracellular parasite *Toxoplasma gondii* infection controlling amino acid levels

**DOI:** 10.1128/msphere.00369-24

**Published:** 2024-07-09

**Authors:** Matthew D. White, Rajendra K. Angara, Leticia Torres Dias, Dhananjay D. Shinde, Vinai C. Thomas, Leonardo Augusto

**Affiliations:** 1 Department of Pathology, Microbiology, and Immunology, University of Nebraska Medical Center, Omaha, Nebraska, USA; 2 Program in Health Science, University of Santo Amaro (UNISA), São Paulo, Brazil; 3 Cognitive Neuroscience of Development & Aging Center, University of Nebraska Medical Center, Omaha, Nebraska, USA; University at Buffalo, Buffalo, New York, USA

**Keywords:** *Toxoplasma gondii*, endoplasmic reticulum, amino acid, behavior, ER-phagy

## Abstract

*Toxoplasma gondii,* a widespread parasite, has the ability to infect nearly any nucleated cell in warm-blooded vertebrates. It is estimated that around 2 billion people globally have been infected by this pathogen. Although most healthy individuals can effectively control parasite replication, certain parasites may evade the immune response, establishing cysts in the brain that are refractory to the immune system and resistant to available drugs. For its chronic persistence in the brain, the parasite relies on host cells’ nutrients, particularly amino acids and lipids. Therefore, understanding how latent parasites persist in the brain is crucial for identifying potential drug targets against chronic forms. While shielded within parasitophorous vacuoles (PVs) or cysts, *Toxoplasma* exploits the host endoplasmic reticulum (ER) metabolism to sustain its persistence in the brain, resulting in host neurological alterations. In this study, we demonstrate that *T. gondii* disrupts the host ER homeostasis, resulting in the accumulation of unfolded protein within the host ER. The host counters this stress by initiating an autophagic pathway known as ER-phagy, which breaks down unfolded proteins into amino acids, promoting their recycling. Our findings unveil the underlying mechanisms employed by *T. gondii* to exploit host ER and lysosomal pathways, enhancing nutrient levels during infection. These insights provide new strategies for the treatment of toxoplasmosis.

**IMPORTANCE:**

Intracellular parasites employ several mechanisms to manipulate the cellular environment, enabling them to persist in the host. *Toxoplasma gondii*, a single-celled parasite, possesses the ability to infect virtually any nucleated cell of warm-blooded vertebrates, including nearly 2 billion people worldwide. Unfortunately, existing treatments and immune responses are not entirely effective in eliminating the chronic persisting forms of the parasite. This study reveals that *T. gondii* induces the host’s autophagic pathway to boost amino acid levels in infected cells. The depletion of amino acids, in turn, influences the persistence of the parasite’s chronic forms. Significantly, our investigation establishes the crucial role of host endoplasmic reticulum (ER)-phagy in the parasite’s persistence within the host during latent infection.

## INTRODUCTION

Apicomplexan parasites are among the most widespread, infecting both humans and animals on a global scale. In humans, they contribute to a spectrum of diseases with significant mortality rates, impacting billions of people worldwide. Malaria, cryptosporidiosis, and toxoplasmosis stand out as the primary diseases caused by parasites in this phylum (1). These parasites effectively manipulate the infected cells to create the necessary niche for establishing the infection (2–5). *Toxoplasma gondii* has globally infected nearly 2 billion people due to its capability to invade virtually any nucleated cell in warm-blooded animals, leading to the development of toxoplasmosis (6). Despite its high infection success rate, this parasite relies on host nutrients to survive. It is auxotrophic for a variety of essential components such as amino acids, lipids, and metabolites, which play a crucial role in establishing the infection (5, 7–9). To establish a persistent infection within host cells, *T. gondii* coordinates the pathways, metabolism, and organelles of infected cells to acquire nutrients (10–14). Within the parasitophorous vacuole (PV), replicative forms (tachyzoites) have the ability to recruit infected cell organelles such as mitochondria, lysosome, and endoplasmic reticulum (ER) to acquire nutrients (9, 15, 16). Notably, *T. gondii* uses the effector protein MAF1 to anchor host mitochondria, facilitating the hijacking of lipids (2, 9, 16). Furthermore, *T. gondii* coordinates the protein synthesis of infected cells to enhance arginine levels to acquire this and other amino acids through the Apicomplexan Amino Acid Transporter family (ApiATs) (10, 17). In addition to utilizing amino acid transporters, *T. gondii* hijacks the host endosomal complexes required for transport machinery to internalize cytosolic host proteins. These proteins are subsequently incorporated into the parasite’s vacuolar system for degradation (7, 11, 18, 19).

After invasion, *T. gondii* recruits the host ER to facilitate its persistence during infection (3, 5). However, the specific molecular mechanisms and key *T. gondii* proteins involved in these processes have yet to be fully identified. Surprisingly, we have previously shown that through this interaction, *T. gondii* co-opts the host ER and Unfolded Protein Response (UPR) proteins to facilitate the spread of the infection throughout the body (20). The high-affinity association between the host ER and *T. gondii* is mediated by unknown effector protein(s), as the protein responsible for recruiting and anchoring the host ER to the parasitophorous vacuole membrane (PVM) remains unidentified. However, it has been shown that the effector protein ROP18 interacts with host ER proteins: ATF6β, MOSPD2, and RTN1-C. This interaction plays a significant role in controlling the infected cell and can contribute to the development of encephalitis in mice (3, 21, 22). In addition, the secreted ROP18 phosphorylates RTN1-C in the ER, initiating ER stress-associated apoptosis through the expression of CHOP protein (22).

The intimate association between *Toxoplasma*’s PV membrane (PVM)/cyst wall and the host ER induces stress in this organelle, consequently disrupting the host ER homeostasis in infected cells (20). Given the pivotal role of ER metabolism in various cellular functions, such as autophagy, lipid synthesis, and protein folding, disruptions in ER can significantly impact overall cellular health and function (23–25). While *T. gondii* successfully invades host cells, it lacks the machinery to synthesize specific amino acids essential for its survival and propagation. As a result, the parasite manipulates the host’s ER metabolism to acquire these crucial nutrients, effectively commandeering the host cell’s resources. This approach ensures the parasite’s continual existence within the host environment. Understanding how *T. gondii* exploits host ER metabolism to access amino acids and other nutrients not only sheds light on the intricate dynamics of host-parasite interactions but also lays the foundation for potential therapeutic approaches targeting this vulnerability.

Although it has been demonstrated that *T. gondii* induces autophagy in host cells (14), facilitating potential access to a source of amino acids and nutrients during infection, the precise molecular mechanisms driving this process are not yet fully understood. Considering the critical role of amino acid availability, especially arginine and tryptophan, in facilitating parasite replication and coordinating the parasite’s transition into the chronic stage (26, 27), our aim is to elucidate mechanisms employed by the parasite to regulate the ER metabolism of the infected cell for nutrient acquisition. Autophagic processes have been characterized to facilitate the elimination of unfolded proteins during ER stress and to maintain ER homeostasis. This mechanism, known as ER-phagy, averts the accumulation of unfolded proteins in the ER lumen, thereby preventing cell death (23, 25). During ER-phagy, the ER is sequestered within an autophagosome, which subsequently fuses with a lysosome to form an autolysosome (23, 28). Within the autolysosome, ER proteins undergo breakdown by lysosomal enzymes, leading to the release of available molecules. However, it is still unclear whether the intracellular parasite *T. gondii* exploits this process to enhance nutrient acquisition. This study addresses the mechanism by which *T. gondii* disrupts host ER homeostasis and folding capacity to elevate lysosomal amino acid levels in infected cells. We demonstrate that, throughout infection, in response to the accumulation of unfolded proteins in the host ER, infected cells amplify ER-phagy. This process leads to increased levels of amino acids, including arginine, proline, and isoleucine/leucine, within the host lysosome. As a consequence, the depletion of these amino acids, which gather in the lysosomes, directly impacts the viability of chronic forms. Moreover, in the absence of amino acid intake, normal behavior is reinstated in infected mice, thereby mitigating the infection’s impact on the brain. These findings emphasize a novel role for the autophagic pathway, ER-phagy, in regulating amino acid availability during *T. gondii* infection. This mechanism plays a crucial role in enabling parasites to effectively compete with host cells for limited nutrient resources.

## RESULTS

### *T. gondii* infection disrupts host ER folding capacity

**Fig 1:**
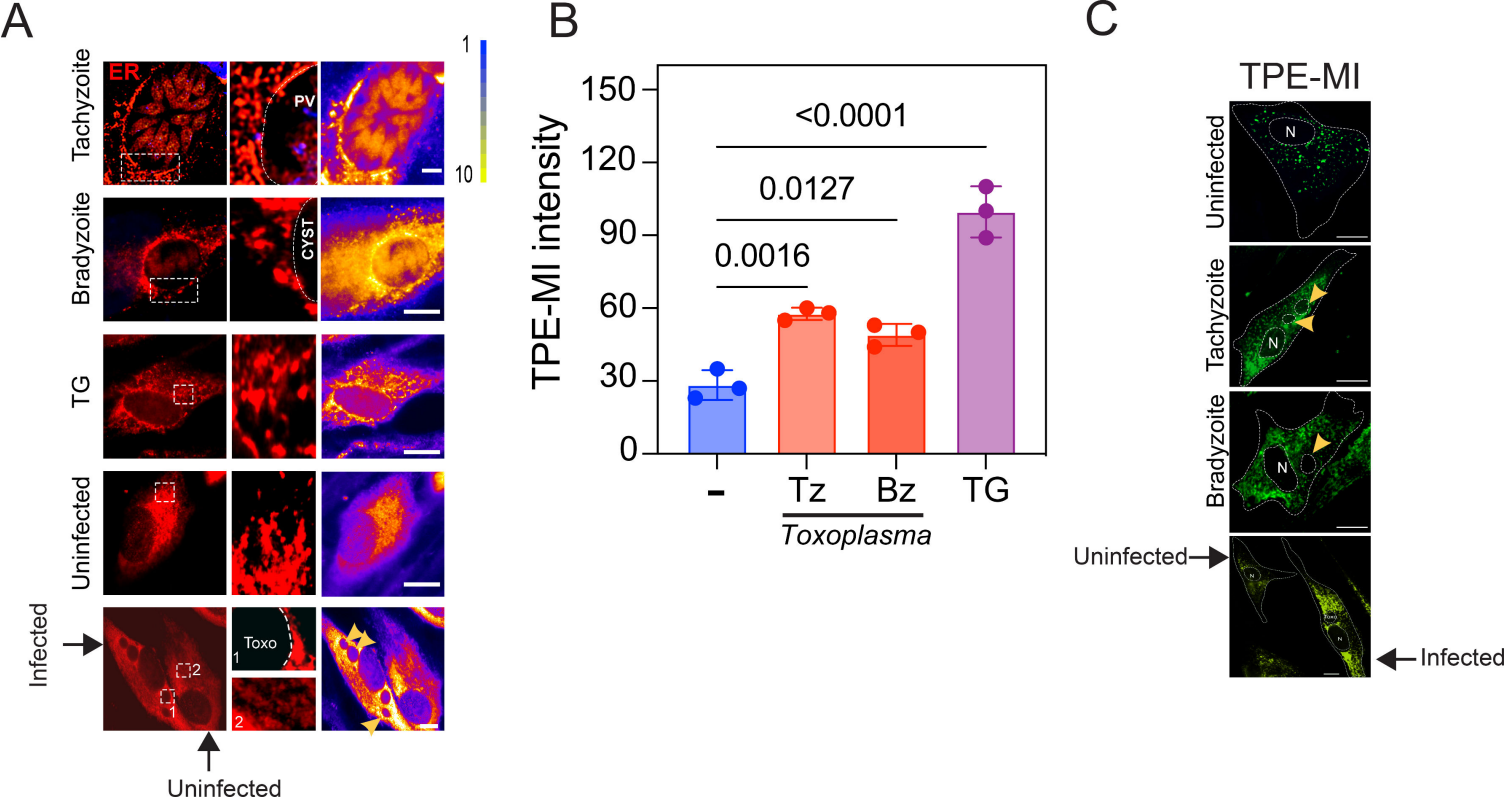
*T. gondii* recruits host ER in both acute and chronic phases. (**A**) Representative images of tachyzoite and bradyzoite-infected cells. Infected cells were probed with ER-Tracker for live-cell imaging to stain the ER (red). Of note, ER-Tracker effectively labeled the *T. gondii* endoplasmic reticulum. The ER-Tracker is shown as a heat map with yellow showing the highest ER-Tracker intensity and blue showing the lowest ER-Tracker intensity. As control: uninfected and thapsigargin (TG)-treated cells (1 mM for 6 h). Scale bar = 5 µm. The last row shows infected (arrowhead) and uninfected cells in the same image. (**B**) Cells were incubated with tetraphenylethene maleimide (TPE-MI) for 10 min. TPE-MI intensity was measured in uninfected (−) and infected cells during the Tz and Bz stages in the channel of the ibidi μ-slide and normalized by the ER area using ImageJ. As a positive control, uninfected cells were treated with 1 mM of TG for 6 h, an ER stress inducer. At least 50 cells were imaged per condition for each of the three independent experiments. ±SD, *n* = 3. (**C**) Representative image of uninfected and infected (tachyzoite and bradyzoite) cells incubated with TPE-MI. Scale bar = 5 µm. N = nucleus of host cell; and Arrow or Toxo = *T. gondii* vacuole.

*T. gondii* secludes itself from the host cytoplasm within the PV forming a niche where it manipulates the host cell through secretory mechanisms, co-opting host organelles such as the host ER. This interaction results in the association of *Toxoplasma* PVM with the host ER membrane (5). First, we confirmed the proximity of the host ER with the *Toxoplasma* vacuole during the infection period, by utilizing an ER-Tracker in live-cell imaging to stain the ER (red). To do so, we infected Human foreskin fibroblasts (HFF) with the cystogenic strain Pru for 24 h. Next, the tachyzoites were induced to differentiate into bradyzoites by incubating infected cells with alkaline media and depriving them of CO_2_. At 24 hours post-infection (hpi) with tachyzoites (Tz) and 4 days post-cyst formation with bradyzoites (Bz), cells were incubated with the ER-Tracker for 1 h. In addition, we show infected (arrowhead) and uninfected cells in the same image. Our data suggest that the host ER surrounds the parasite vacuole at both stages of infection (Fig. 1A).

Given that one of the functions of the ER is to facilitate and coordinate the folding process of proteins recently translated within the ER lumen (23, 29, 30), we aimed to elucidate whether the association or proximity between *T. gondii* and host ER dysregulates the host ER folding capacity. We previously demonstrated that *T. gondii* infection induces host UPR activation (20). However, in this study, we were able to measure the levels of unfolded proteins in infected cells. Accordingly, we incubated infected cells with the thiol probe tetraphenylethene maleimide (TPE-MI), which binds to cysteines of unfolded proteins and becomes fluorescent. Using live-cell microscopy, we measured the TPE-MI intensity exclusively within the ER region by transfecting cells with the ER-resident protein (KDEL) tagged with red fluorescent protein (RFP) (red) to establish its co-localization with ER staining. This methodology enabled us to differentiate and quantify the host ER TPE-MI levels, effectively removing the contribution of parasite and cytosolic background TPE-MI intensity from our measurements. At the 24 hpi (Tz) and 4 days after cyst formation (Bz), cells were exposed to TPE-MI for 10 min. Our findings indicate a higher intensity of TPE-MI within the ER of *Toxoplasma*-infected cells compared to uninfected cells (Fig. 1B; Fig. S1). To further confirm that TPE-MI intensity was associated with the accumulation of unfolded protein and ER stress, cells were treated with the ER stress inducer thapsigargin (TG) for 6 h, revealing an even higher TPE-MI intensity in the ER (Fig. 1B and C; Fig. S1). Our results indicate that only infected cells displayed high levels of TPE-MI intensity (Fig. 1C). This increased intensity was not observed in neighboring uninfected cells. Collectively, our results suggest that *T. gondii* recruits and maintains the interaction with the host ER during both the replicative and dormant stages of infection, significantly enhancing unfolded protein levels in the host ER.

### Host ER-phagy is enhanced in *Toxoplasma*-infected cells

**Fig 2:**
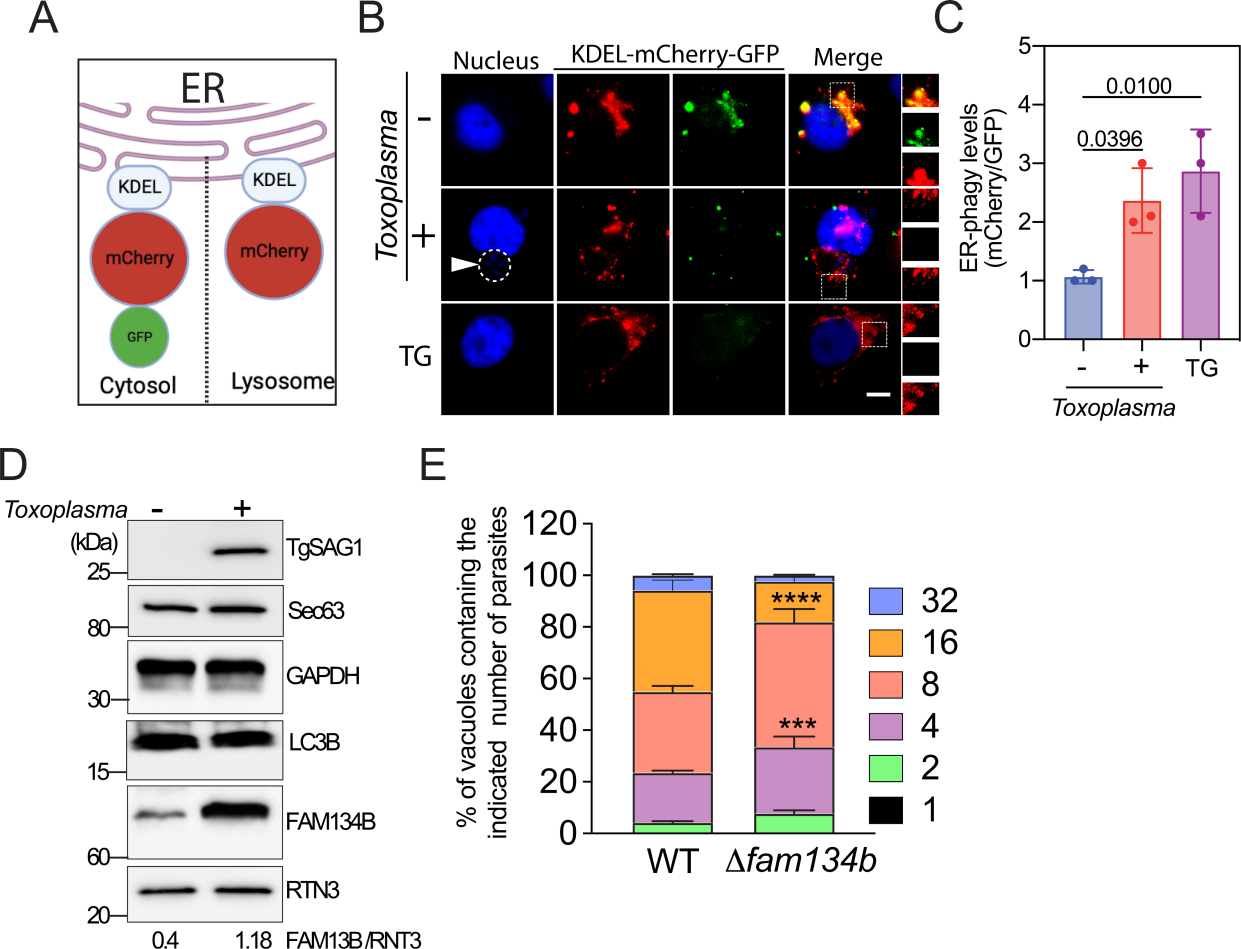
ER-phagy is induced in *Toxoplasma*-infected cells. (**A**) Schematic representation of the plasmid used to transfect cells: ER-resident protein (KDEL) tagged with mCherry and GFP. Upon ER-phagy, the ER protein is integrated into lysosomes, causing instability of GFP in acidic pH, and only the mCherry signal can be detected, whereas in unstressed cells, the protein faces the cytosol, and both GFP and mCherry signals are detected. (**B and C**) Transfected cells were infected with *T. gondii* and after 30 hpi, the GFP and mCherry foci were quantified by live cell imaging of 50 cells per experiment. The data are presented as the mean ± SD, *n* = 3. The arrow indicates PV. (**D**) Then, cells were harvested and the levels of total FAM134B, RTN3, Sec63, TgSAG1 (*T. gondii*), and GAPDH were measured by immunoblot analyses. The pixel intensities of the bands were determined, and the ratio FAM134B/RNT3 is presented. (**E**) Parasite doubling assay. At the indicated time points, the number of parasites in 100 random vacuoles was plotted as a percentage of the total number of vacuoles examined. ±SD, *n* = 3. **, *P* < 0.01; ***, *P* < 0.005.

Cells have evolved distinct mechanisms to alleviate the accumulation of unfolded proteins in the ER lumen through the activation of UPR (31). Consequently, this leads to reprogramming of gene expression and translation. Remarkably, prolonged and persistent ER stress can trigger cell death. However, to prevent damage, cells can reinforce the ER stress response by activating the specialized autophagic pathway to degrade and reduce the levels of unfolded proteins in the ER (23, 29, 32, 33). To investigate whether cells undergo ER-phagy in response to ER stress during *T. gondii* infection, cells were transfected with the ER-resident protein (KDEL) tagged with green fluorescent protein (GFP) (green) and mCherry (red), which faced the cytosol. Subsequently, the cells were infected with *T. gondii*. During ER-phagy, the ER protein is targeted to autophagosomes/lysosomes, where the acidic pH destabilizes GFP, as described previously (34), resulting in the detection of only the mCherry signal (Fig. 2A). Since we observed unfolded protein accumulation in the host ER at 24 hpi, we aimed to understand the consequences and host responses. At 30 hpi, we quantified the number of cells exhibiting punctate signals with both mCherry and GFP or only mCherry signals to determine if infected cells undergo ER-phagy. As a positive control, we used an ER stress inducer (TG). The significant increase in mCherry signal in *Toxoplasma*-infected cells provides evidence suggesting that these cells undergo ER-phagy (Fig. 2B and C). In addition, we examined the GFP loss of signal at different time points of infection (Fig. S2). Considering that ER-phagy is facilitated by FAM134B within the ER (23), we proceeded to assess the protein levels of FAM134B in the infected cells. Our findings indicate an increase in FAM134B expression, suggesting that the infected cells undergoing ER-phagy (Fig. 2D), but we did not observe significant changes in other ER and general autophagy markers, such as RTN3 and LC3, respectively. Together, our results suggest that *T. gondii* infection induces ER stress and accumulation of unfolded protein in the ER lumen. In addition to UPR activation, infected cells enhance ER-phagy to alleviate ER stress.

To gain deeper insights into the role of FAM134B and ER-phagy in the infection, we employed CRISPR/Cas9 technology to disrupt the FAM134B gene in cells. The absence of FAM134B resulted in a notable delay in parasite replication. This finding strongly implies the critical importance of activating ER-phagy during *T. gondii* infection.

### Host lysosome proteolytic activity is enhanced during *T. gondii* infection

**Fig 3:**
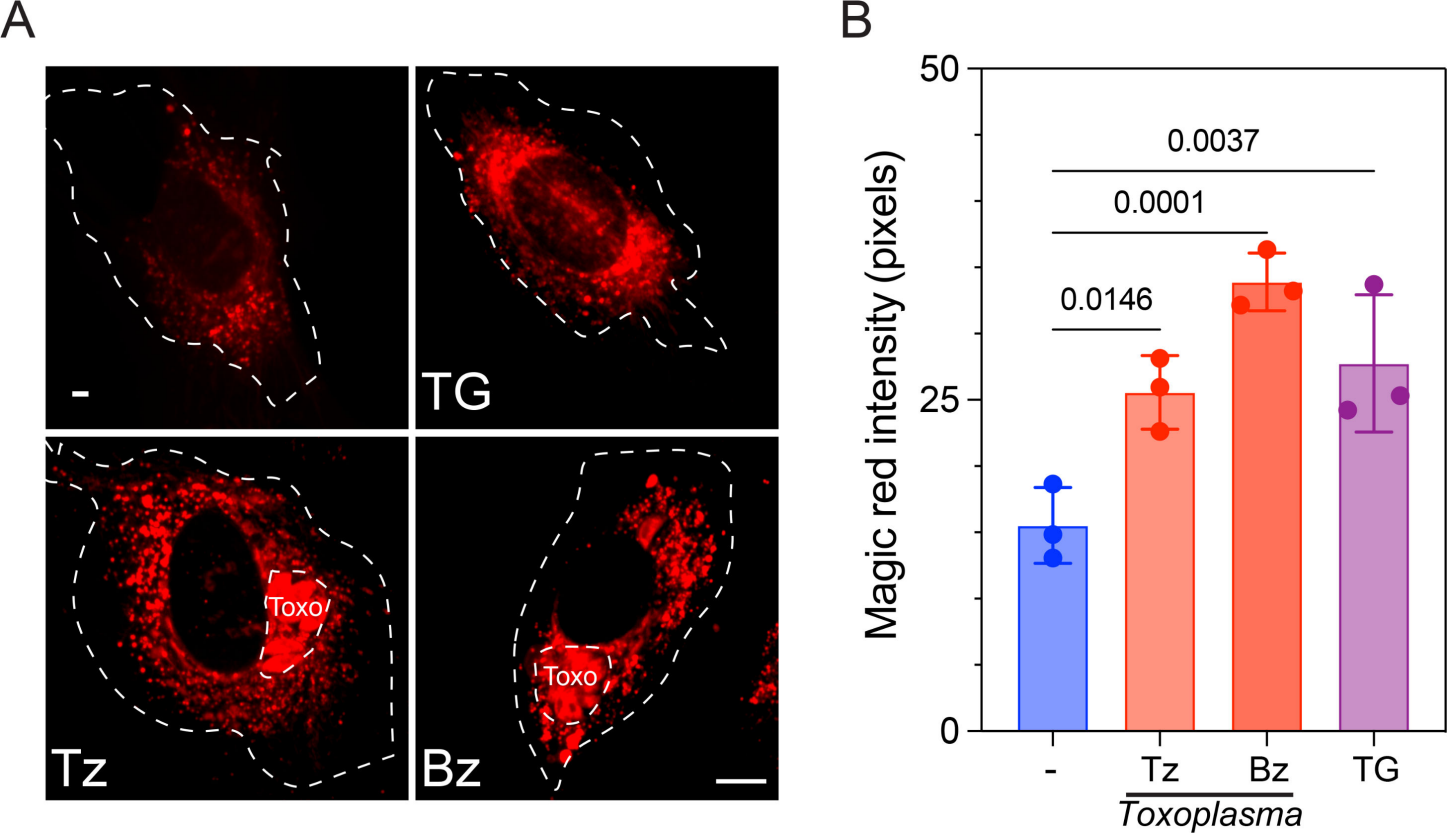
Host lysosomal proteolytic activity increases during *T. gondii* infection. (**A and B**) Quantification of Magic Red reveals that lysosomes are more proteolytically active in both Tz- and Bz-infected cells compared to uninfected cells (−). As control: uninfected and TG-treated cells (1 mM for 6 h). The intensity of Magic Red (pixels) was determined by live cell imaging and quantified using ImageJ. The values were normalized to cell area and uninfected cells at each time point. The parasite (Toxo) intensity was excluded from quantification. The data are presented as the mean ± SD, *n* = 3. Scale bar = 5 µm.

Given that *T. gondii* infection induces ER-phagy in infected cells, we aimed to investigate whether there is an increase in proteolytic activity and degradation within the host lysosome during the infection. To accomplish this, we assessed the proteolytic activity of the lysosomal protease cathepsin B in both uninfected and infected cells, utilizing a fluorescence-based Magic Red assay. The Magic Red assay utilizes a cathepsin B peptide substrate capable of permeating the cell membrane (35). Upon enzymatic cleavage by cathepsin B, cresyl violet generates red fluorescence, with the fluorescence intensity intensifying as the enzymatic activity progresses. Using live cell confocal microscopy, we examined lysosomal cathepsin B activity in both uninfected and *Toxoplasma*-infected cells. As our analysis revealed a higher fluorescence intensity in infected cells and considering that cathepsin B activity is directly linked to acidic lysosomal pH (35), our data strongly suggest elevated cathepsin B activity throughout the infection, implying concurrent lysosomal acidification (Fig. 3A and B). Interestingly, the levels of TG-treated cells are higher compared to uninfected cells, suggesting that somehow ER stress leads to changes in lysosomal metabolism, either driven by *Toxoplasma* or an ER stress inducer. It is important to note that the parasite intensity is excluded from quantification.

### *T. gondii* infection enhances lysosomal amino acid levels in the host

**Fig 4:**
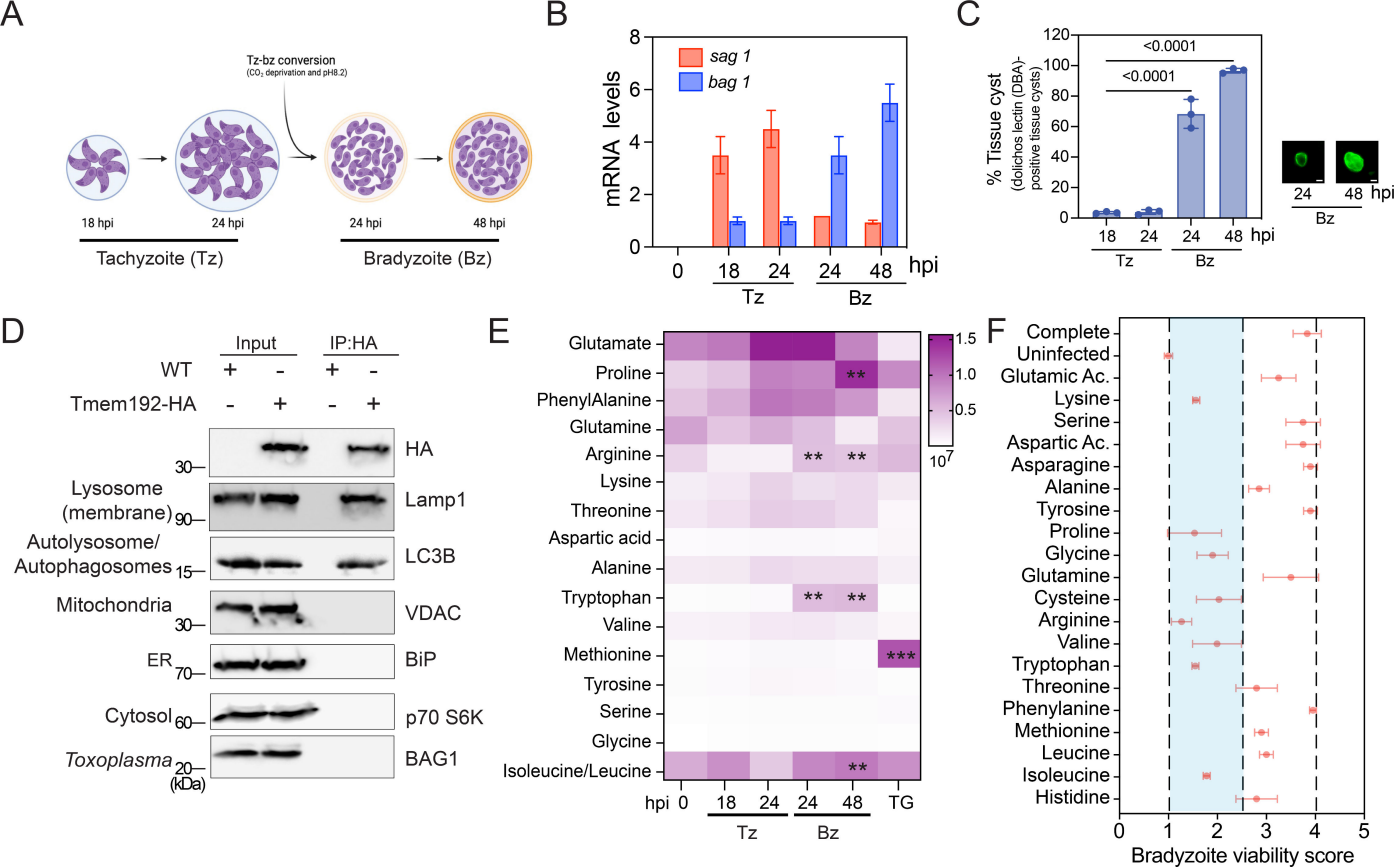
*T. gondii* infection induces host lysosomal amino acid accumulation. (**A**) LYSO-IP cells were infected with *T. gondii* and at the indicated time points the lysosomes were isolated. (**B**) Stage-specific markers were used to confirm tachyzoite (*sag1*) and bradyzoite (*bag1*) stages during the experimental design by reverse transcription quantitative real-time (RT-qPCR), and expression levels of both genes were normalized to *Tg-gapdh*. The basal levels were considered as 1, serving as the baseline reference for comparison. (**C**) The percentage of cyst formation was determined in 100 cells using Dolichos lectin conjugated to rhodamine to visualize the cyst wall. Dolichos lectin conjugated to rhodamine staining was used to confirm cyst wall formation and representative images of the cyst wall. Scale bar = 5 µm. (**D**) Host lysosomes were isolated by immunoprecipitation using HA-magnetic beads and confirmed by immunoblotting using specific organelle markers, as indicated. (**E**) Lysosomal fractions were analyzed by mass spectrometry, and the levels of amino acids were determined. The values were normalized to uninfected cells at each time point, as control we use TG treatment. The data are presented as the mean ± SD, *n* = 3. (**F**) Amino acids were individually depleted from the media and used to evaluate bradyzoite viability. After confirmation of cyst formation using Dolichos, cyst walls were lysed, and the parasites were separated from the host cell debris by filtering. Parasites were counted and quantified by RT-qPCR. Five hundred parasites were used to infect a HFF monolayer. After 12 days, parasite viability was determined by plaque assay. The bradyzoite viability score was determined by the percentage of plaques number relative to completed media. The data are presented as the mean ± SD, *n* = 3.

As ER-phagy is an autophagic process that selectively recycles ER proteins through lysosomes, and the given enhanced lysosomal proteolytic activity during infection, our next question was whether elevated host ER-phagy leads to the accumulation of amino acids in lysosomes. To investigate this, we used the LYSO-IP method, an unbiased and established technique for studying lysosomal metabolism. We generated a stable LYSO-IP cell line by inserting a copy of TMEM-192-HA (HA = hemagglutinin tag) into the genome. TMEM-192, a lysosomal protein, facilitated the rapid isolation of lysosomes from the host cytosol by HA immunoprecipitation using magnetic beads, as described (36). Next, LYSO-IP cells were infected with the *Toxoplasma*-Pru strain, and we harvested the cells at 18 and 24 hpi during replicative tachyzoites (Tz) infection; or at 24 and 48 hours post-bradyzoites differentiation (Bz) (Fig. 4A). Notably, we confirmed the expression of stage-specific genes for tachyzoites and bradyzoites, as well as the formation of cyst wall by identifying positive cysts using Dolichos lectin conjugated to rhodamine (Fig. 4B and C). Next, the isolation of lysosomes was confirmed by western blot using organelles-specific antibodies (Fig. 4D; Fig. S3). The lysosome fractions were collected and subjected to tandem mass spectrometry for the analysis of amino acid levels. Interestingly, we observed a time-dependent enrichment of several amino acids in lysosomes post-infection when compared to mock-infected cells at each time point. Surprisingly, at 48 h during bradyzoite infection (Bz 48 h), the lysosomal levels of arginine, proline, lysine, isoleucine/leucine, and tryptophan showed a significant increase compared to replicative tachyzoites (Fig. 4E). Importantly, all results were normalized to mock-infected cells at each time point. In addition, upon TG treatment, we found a predominant accumulation of methionine, suggesting that the accumulation of other amino acids may be driven by *Toxoplasma* infection.

Amino acids accumulated in the lysosomes due to protein degradation are transported to the cytoplasm by specific permeases (37–39), where they can be reused in anabolic processes. Since *T. gondii* is an auxotroph for several amino acids, including arginine (26), and the depletion of arginine significantly impairs parasite replication (40), we have investigated the impact of the availability of different amino acids on the viability of bradyzoites. To do so, cells were infected with the *Toxoplasma-*Pru strain and after cyst formation, cells were cultured for 4 days in a medium depleted of individual amino acids [supplemented with dialyzed fetal bovine serum (FBS)]. Subsequently, the viability of bradyzoites within the cysts was assessed through pepsin digestion, followed by infecting a cell monolayer and counting the plaques. Briefly, cysts were released from infected cell layers using mechanical techniques and pepsin digestion. Parasite counts were established, and an equal number of parasites were used to infect a cell monolayer. Additionally, a portion of pepsin-treated parasites underwent genomic DNA purification for quantitative polymerase chain reaction (qPCR), utilizing specific primers to quantify parasite genomes per microliter. After a 12-day incubation period, plaques originating from bradyzoites were enumerated with a light microscope. The plaque count was normalized to the initial genome count, thus assessing bradyzoite viability. Bradyzoite viability in infected cells treated with the completed amino acid media was considered 100%, and the other media conditions were compared to it. Our data indicate that the depletion of arginine, proline, glycine, isoleucine, valine, lysine, tryptophan, or cysteine significantly decreases bradyzoite viability (Fig. 4F; Fig. S4). This information underscores the critical role of amino acid availability in the context of chronic *T. gondii* infection.

## DISCUSSION

Intracellular pathogens typically sequester themselves within vacuoles, which subsequently fuse with host cell organelles to obtain essential nutrients necessary for establishing infection (8, 41). For instance, *T. gondii* parasites inhabit a nonfusogenic PV that closely associates with host organelles, such as the ER and mitochondria. Additionally, *T. gondii* can co-opt host endosomal vesicles and cytosolic proteins to acquire nutrients (5, 42–44). The recruitment of host organelles to the PV is thought to enable *T. gondii* to modulate crucial host cell functions, including antigen presentation, nutrient production, and the suppression of apoptosis (4, 5).

In this study, we aimed to investigate the intricate mechanisms underlying the connection between the *T. gondii* vacuole and the host ER, specifically elucidating how this interaction enhances amino acid availability to support parasite survival. It is noteworthy that this association persists even under pharmacological induction of ER stress (45). However, the role of ER homeostasis in bradyzoite viability is not yet fully understood. Our findings demonstrate a significant link between the infection and the ER, resulting in an increased presence of unfolded proteins within the ER lumen of infected cells. This, in turn, prompts infected cells to activate the UPR (20) and initiate ER-phagy, which increases available amino acid pools. Surprisingly, induction of ER stress by *Toxoplasma* or a pharmacological ER stress inducer has different outcomes in lysosomal amino acid metabolism, even though cathepsin activity is enhanced in both scenarios. As a result, lysosomes in infected cells differentially accumulate several amino acids, potentially available for the parasite’s use. The outstanding question centers on how the parasite accesses these lysosomal amino acids. One potential mechanism involves incorporating these nutrients into the vacuole through a process similar to that observed with Rab-positive vesicles (42, 44). Alternatively, *T. gondii* may directly interact with lysosomes, inducing nutrient transport to the cytosol or causing lysosomal damage to gain access to these nutrients. Our findings highlight the importance of specific amino acids in maintaining bradyzoite viability during the chronic phase *in vitro*. However, the precise mechanism by which *T. gondii* acquires these lysosomal metabolites or enhances nutrient availability within infected cells to ensure its survival remains an elusive aspect that requires further exploration.

*T. gondii* is known to be auxotrophic for several amino acids including arginine and tryptophan (4, 26). However, our study emphasized the significance of amino acids such as proline, which were found to be crucial for maintaining the viability of the parasite in the cyst *in vitro*. However, it is not clear yet the role of starvation of this amino acid in parasite differentiation into chronic forms and cyst formation. In an animal model, amino acid levels in the brain changed upon *Toxoplasma* infection; however, it is unknown how these changes impact bradyzoite viability and the immune response against tissue cysts or infected neurons. It is known that neurotransmitter levels and excitatory or inhibitory synapses depend on cellular metabolism in neurons, astrocytes, and microglia. These metabolic changes can affect the neurological alterations caused by the infection and even lead to recrudescence. Moreover, the effects of a restricted amino acid diet or changes in nutrient levels are not fully understood in the context of *Toxoplasma* infection. Some data suggest that gut microbiome composition is not drastically altered during chronic *Toxoplasma* infection (45), while other studies indicate that alterations in gut microbiota contribute to cognitive deficits induced by chronic *Toxoplasma* infection (46). Therefore, the relationship between nutrient availability, diet, gut microbiota, cognitive or neurological alterations, parasite numbers, and pathogenicity remains unclear.

It is noteworthy that not only specific nutrients such as amino acids, but also lipids, can significantly influence *T. gondii* pathology in the brain. A crucial aspect of future research is understanding how various nutrients and metabolites collectively contribute to neuropathology during chronic infection. Therefore, understanding the metabolic dependence of bradyzoites and their host during latent infection, as well as the factors enabling parasite persistence, is crucial for the development of new therapies and the elimination of the parasite.

## MATERIALS AND METHODS

### Host cell and parasite culture

*T. gondii* parasites [Prugniaud (Pru) strain-type II] were propagated in human foreskin fibroblast cells (HFF, ATCC) using Dulbecco’s modification of Eagle’s medium (DMEM) supplemented with 10% heat-inactivated FBS and penicillin-streptomycin. The parasites were obtained from chronically infected BALB/cJ mice. HeLa and human embryonic kidney (HEK293) cells were cultured in DMEM supplemented with 10% FBS and penicillin-streptomycin at 37°C with 5% CO_2_. Infection was performed using a multiplicity of infection (MOI) of 3 with the Pru strain for the indicated time points. The tachyzoite-bradyzoite conversion was performed by incubating infected cells with alkaline media and deprivation of CO_2_.

Disruption of the FAM134B gene in HFF was carried out using the CRISPR/Cas9 method. Two distinct sgRNAs were designed using the Integrated DNA technology tool (g1-GTGCACATTTTTTACGATCT and g2-AGGTATCCTGGACTGATAAT). The sgRNAs were prepared using the EnGen sgRNA synthesis kit (New England BioLabs), along with an sg control (g-control-CATCCTCGGCACCGTCACCC). The sgRNAs were then associated with EnGen Spy Cas9 NLS protein (New England BioLabs) at room temperature for 15 min. Cells were then transfected with the guide bound to Cas9 protein using the Lipofectamine CRISPRMAX Cas9 transfection reagent (Thermo Fisher Scientific). After culturing the transfected cells for 48 h, 800 cells were validated by immunoblotting using FAM134B-specific antibody (Table 1).

**TABLE 1:** Antibodies information^*a*^

Antibody	Company (cat. number)	Dilution
FAM134B	Cell Signaling-Rabbit mAb #83414	1:1,000
GAPDH	Cell Signaling-Rabbit mAb #5174	1:1,000
HA	Cell Signaling-Rabbit mAb #3724	1:1,000
LAMP1	Cell Signaling-Rabbit mAb #9091	1:1,000
LC3B	Cell Signaling-Rabbit mAb #3868	1:1,000
VDAC	Cell Signaling-Rabbit mAb #4661	1:1,000
p70 S6Kinase	Cell Signaling-Rabbit mAb #2708	1:1,000
SEC63	Abcam- ab183046	1:1,000
RNT3	Thermo Fisher-10A8	1:1,000
Anti-mouse IgG, HRP-linked Ab	Cell Signaling-#7076	1:500
Anti-rabbit IgG, HRP-linked Ab	Cell Signaling-#7074	1:500

^*a*^ List of antibodies with company and dilution information.

For doubling assays, tachyzoites were allowed to invade an HFF host cell monolayer. After 2 h, the uninvaded parasites were removed by replacing the medium with the medium. At 12, 24, and 36 hpi, cells were fixed with 4% paraformaldehyde for 20 min and the number of parasites in 100 randomly selected vacuoles was then counted.

### TPE-MI staining

To prepare a stock solution of TPE-MI, TPE-MI was dissolved in DMSO at a concentration of 2 mM. HFF cells were plated in ibidi-treated channel μ-slide VI0.4 (1  ×  10^4^ cells per channel; ibidi) and allowed to adhere overnight. Cells were infected with *Toxoplasma* (Pru strain) at a MOI of 3 for 2 h and then washed with medium to remove extracellular parasites. After 24 h, infected cells were incubated with freshly diluted TPE-MI (50 µM in media) for 10 min at 37°C. The TPE-MI solution was then washed out with media, and Z-stacked confocal images were obtained for each cell using a Nikon Eclipse Ti2 spinning disc microscope within an environmental chamber (5% CO_2_ and 37°C) with a 60× oil immersion objective and an Okolab Bold Line stage top incubator. The fluorescence intensity of TPE-MI in each cell was normalized by the cell area using ImageJ software. To achieve this, cell margins and the PV were delineated by drawing lines. Subsequently, a colocalization image was generated using Image J software, and the intensity of TPE-MI was measured utilizing the same software, as described in reference (47). The intensities of both uninfected and infected cells were measured within the same channel as well as in separate channels of the ibidi μ-slide. At least 50 cells were imaged per condition for each of the three independent experiments.

### Cathepsin B activity

Cathepsin B was measured using Magic Red as previously described (35). Briefly, HFF were plated in ibidi-treated channel μ-slide VI0.4 (1  ×  10^4^ cells per channel; ibidi) and allowed to adhere overnight. Cells were infected with *T. gondii* (Pru strain) at a MOI of 3 for 2 h and then washed with medium to remove extracellular parasites. At indicated time points, tachyzoite or bradyzoite parasites were visualized, then uninfected and infected cells were incubated with 50 µL diluted Magic Red (ImmunoChemistry Technologies) in phenol-red free DMEM and incubated for 30 min at 37°C and 5% CO_2_, and cells were live imaged, with identical capture settings, using z-stacks of 0.3 µm steps with a Nikon Eclipse Ti2 spinning disc microscope within an environmental chamber (5% CO_2_ and 37°C). The Magic Red fluorescence intensity was measured, using ImageJ software, and normalized to the cell area, excluding the parasite intensity. At least 20 cells were measured per condition in each of the three independent experiments.

### ER-phagy levels

HFF (3  ×  10^4^ cells per well of a 24-well plate or 1  ×  10^5^ cells per well of a 6-well plate) were reverse transfected with 0.5 µg of pCW57-CMV-KDEL-mCherry-GF using Fugene HD (Promega) according to the manufacturer’s instructions. After 24 h post-transfection, cells were replated in ibidi-treated channel μ-slide VI0.4 (1  ×  10^4^ cells per channel; ibidi), and infected with *T. gondii* for 2 h (MOI, 3). Cells were live imaged, with identical capture settings, using z-stacks of 0.3 µm steps with a Nikon Eclipse Ti2 spinning disc microscope within an environmental chamber (5% CO_2_ and 37°C). At least 25 cells were measured per condition in each of the three independent experiments.

### Immunoblot

Infected cells were harvested in RIPA buffer solution supplemented with complete, EDTA-free protease inhibitor cocktail (Roche). Protein quantification was performed using the BCA Protein Assay Kit (Pierce). Equal amounts of protein lysates were separated by 4%–20% Mini-PROTEAN TGX (BioRad), and proteins were transferred to a nitrocellulose membrane. Immunoblot analyses were done using primary antibodies (Table 1) for 18 h diluted in TBS-T 5% milk, followed by secondary antibody horseradish peroxidase (HRP)-conjugated. Proteins in the immunoblot membranes were visualized using the Azure Biosystem C600. The membranes were incubated with Stripping buffer (Restore WB Stripping buffer—Thermo Scientific) to be incubated with subsequent antibodies if needed. Immunoblot analyses were carried out for three independent experiments.

### Lysosome immunoprecipitation

HEK293-TMEM192-HA cells were plated (3  ×  10^6^ cells per 150 mm tissue-culture dishes). After 24 h, cells were infected with *Toxoplasma*-Pru for 2 h (MOI, 3) and then harvested at the indicated time points. The cyst formation was induced by alkaline stress (pH 8.2) combined with CO_2_ deprivation. At the indicated time points, cells were washed three times with cold Potassium-Phosphate-buffered saline (KPBS) (136 mM KCl, 10 mM KH_2_PO_4_, pH 7.3), scraped on ice in 1 mL of cold KPBS, and centrifuged at 900 × *g* for 2 min at 4°C. The pellets were resuspended in 950 µL and 25% of each sample was reserved as a whole-cell fraction, for further processing by liquid chromatography/tandem mass spectrometry (LC/MS/MS) analysis. The remaining cells were gently homogenized with 15 strokes of a 2 mL homogenizer, the lysates were then centrifuged at 900 × *g* for 2 min at 4°C. Next, the supernatant containing the lysosomes was incubated with KPBS prewashed anti-HA magnetic beads (Pierce) for 5 min. Isolated lysosomes were then gently washed five times with KPBS using the DynaMag Spin Magnet. The amino acid and metabolite extraction from lysosomes was performed by incubating the isolated lysosomes with 100 µL of metabolite methanol extraction buffer (80% methanol, 20% water containing internal standards) for 10 min on ice, followed by beads removal. The metabolite extract (liquid fraction) was then centrifuged at 900 × *g* for 5 min at 4°C. The supernatant was collected and analyzed by LC-MS/MS to determine the amino acid levels (Table 2). Mass spectrometry analysis was performed by the Mass-Spectrometry and Proteomics Core Facility at the University of Nebraska Medical Center. 13C15N-labeled canonical amino acid (CAA) mix procured from the Cambridge Isotope Laboratory was used as the internal standard during the sample preparation.

**TABLE 2:** Amino acids measurements^*a*^

Amino acid	Lysosome fraction: amino acid levels [peak area (10^5^)]																	
	Uninfected cells			Tachyzoites at 18 hpi			Tachyzoites at 24 hpi			Bradyzoites at 24 hpi			Bradyzoites at 48 hpi			Uninfected cells (TG treatment)		
	Expt. 1	Expt. 2	Expt. 3	Expt. 1	Expt. 2	Expt. 3	Expt. 1	Expt. 2	Expt. 3	Expt. 1	Expt. 2	Expt. 3	Expt. 1	Expt. 2	Expt. 3	Expt. 1	Expt. 2	Expt. 3
Glutamate	100	85	79	100	110	83	130	150	190	170	150	150	73	84	110	17	18	18
Proline	44	36	23	59	40	32	53	91	130	150	52	57	92	110	230	130	56	70
PhenylAlanine	58	45	30	68	58	54	69	100	130	130	85	69	64	65	100	24	3.6	29
Glutamine	82	66	59	45	45	40	41	63	80	52	59	33	18	16	13	49	45	42
Arginine	38	28	31	11	12	12	12	11	11	58	46	38	44	45	42	55	53	50
Lysine	17	13	9.9	22	16	15	21	34	46	31	22	19	22	24	38	21	8	21
Threonine	24	17	12	27	23	21	27	38	50	51	32	25	24	24	38	4.3	2.6	12
Aspartic acid	2.3	3.3	3.1	4.1	4.3	4	3.7	5.6	5.6	4.7	4.9	4.9	3.9	4.1	4.6	12	4	5.5
Alanine	21	16	11	19	17	14	19	29	37	35	24	18	18	22	36	18	7.8	10
Tryptophan	2.4	2	1	3	2.7	2.2	3.1	4.8	4.3	58	41	52	50	60	48	2.3	2.2	1.8
Valine	12	8.1	7.3	11	10	9.8	12	16	23	23	18	13	9.8	12	18	5.1	8.3	9.7
Methionine	2.6	1.8	1.3	3.4	3	2.8	3.8	5.5	7	7.4	4.8	3.9	3.2	3.1	4.9	83	133	144
Tyrosine	3.2	3.1	1.7	4.2	3.9	3.3	4.8	5.7	9.4	9.8	4.9	4.3	4.4	4.2	6.6	0.73	2.7	3.1
Serine	1.1	0.8	0.93	1.6	1.6	1.4	1.8	2.5	3.8	3.4	2.1	1.7	2.1	1.8	4.4	5.4	2.9	3.3
Glycine	0.49	0.19	0.4	0.55	0.55	0.49	0.64	1.4	1.2	1.6	0.86	0.76	1.2	0.91	1.5	1.1	0.53	0.55
Isoleucine/leucine	78	62	46	94	78	73	92	13	17	160	12	90	75	85	130	77.2	80.5	87.4

^*a*^ Lysosomal fractions were analyzed by mass spectrometry, and the levels of amino acids were determined by liquid chromatography-high-resolution mass spectrometer/tandem mass spectrometry (LC-HRMS/MS).

### Liquid chromatography-high-resolution mass spectrometer/tandem mass spectrometry analysis of metabolites

A high-resolution mass spectrometer (HRMS), specifically the Tribid Orbitrap Exploris 480 (Thermo) connected to an ultra-high-performance liquid chromatography (UHPLC) system, was employed for metabolite analysis. The chromatographic separation was performed by liquid chromatography using XBridge Amide (150 mm × 2.1 mm ID; 1.7 µm particle size) analytical column and a binary solvent system with a flow rate of 0.3 mL/min. Mobile phase A was composed of 10 mM ammonium acetate and 10 mM ammonium hydroxide containing 5% acetonitrile in LC-MS grade water. Mobile phase B was 100% LC-MS grade acetonitrile. The column temperature was maintained at 40°C, while the autosampler was set to 5°C. UHPLC pumps operated in gradient mode, and a 5 µL injection volume was used per sample.

For untargeted metabolomics in data-dependent MS/MS acquisition mode (DDA), the HRMS Orbitrap (Exploris 480) was employed in polarity switching mode. Electrospray ionization parameters were optimized with −3,500V and 4,000V electrospray ion voltage in negative and positive modes, respectively. The ion transfer tube temperature was set to 400°C, and the m/z scan range was 70–1,050 Da. Sheath gas, auxiliary gas, and sweep gas were optimized according to the UHPLC flow rate. Orbitrap resolution for precursor ion as well as for fragment ion scan was maintained at 240,000 and 120,000, respectively. Normalized collision energies at 30%, 50%, and 150% were used for the fragmentation. Data acquisition was performed in profile mode using Xcaliber software (Thermo). The software includes Qual-, Quant-, and FreeStyle browsers, which were utilized for profiling labeled 13C15N-CAA standards in all samples. Identification and detection of metabolites were supported by Compound Discoverer software (Thermo). The HMDB and KEGG databases were integrated for metabolite identifications. The high-resolution mass spectrometry allowed for accurate precursor and fragment ion mass detection, facilitating confident molecular annotation and metabolite assignments.

### Bradyzoite viability

After cyst formation, the cells were cultured for 4 days in RPMI medium with depleted individual amino acids, supplemented with dialyzed FBS. The depleted medium was prepared by adding 19 amino acids to the RPMI medium lacking one amino acid. After the incubation period, cysts were released from infected cells using mechanical techniques such as scraping and syringe lysis with 25-gauge needles. To prevent any host contamination, the material underwent a filtration process twice and was washed in 20 mL of PBS through centrifugation at 800 rpm for 10 min. Subsequently, the material underwent pepsin digestion, following previously described methods (7). Briefly, the material was transferred to a 15 mL conical tube, the well was washed with 5 mL of HBSS, and a pepsin solution was added to the pellet and incubated at 37°C for 30 min. After the incubation period, a stop reaction (94 mM Na_2_CO_3_) was added to neutralize the reaction. Next, a centrifugation step was performed (800 × *g* for 10 min), and the pellet was resuspended in DMEM media. Then, parasite counts were determined, and an equal number of parasites (500) was used to infect an HFF monolayer. Moreover, a subset of pepsin-treated parasites underwent genomic DNA purification for qPCR, using specific primers (B1) (20) to quantify the number of parasite genomes per microliter. Following a 12-day incubation period, plaques were counted using a light microscope. The plaque count was standardized according to the initial genome and parasite counts, thus evaluating the viability of bradyzoites. Bradyzoite viability in infected cells, treated with the complete amino acid medium, was set at 100%, and the other medium conditions were compared against it. Each experiment was performed three times, each with three technical replicates.

### Quantification and statistical analysis

Quantitative data are presented as means and standard deviations and were derived from at least three biological replicates. Statistical significance was determined using a one-way analysis of variance with Tukey’s post hoc test and multiple two-tailed *t*-tests using Graph Prism software 10. The number of biological replicates and *P* values are indicated in figure legends. For immunoblot analyses, the reported images are representative of at least three independent experiments.

## SUPPLEMENTAL MATERIAL

Supplemental figures msphere.00369-24-s0001.pdf Figures S1 to S4.

ASM does not own the copyrights to Supplemental Material that may be linked to, or accessed through, an article. The authors have granted ASM a non-exclusive, world-wide license to publish the Supplemental Material files. Please contact the corresponding author directly for reuse.
