# Supplementary material for: Selective host autophagy is induced during the intracellular parasite Toxoplasma gondii infection controlling amino acid levels
Source: mSphere. 2024 Jul 9;9(7):e00369-24. doi: 10.1128/msphere.00369-24 (PMC11288035; doi:10.1128/msphere.00369-24)
Supplement: Supplemental figures — Figures S1 to S4. [file msphere.00369-24-s0001.pdf]

Supplementary Figure 1

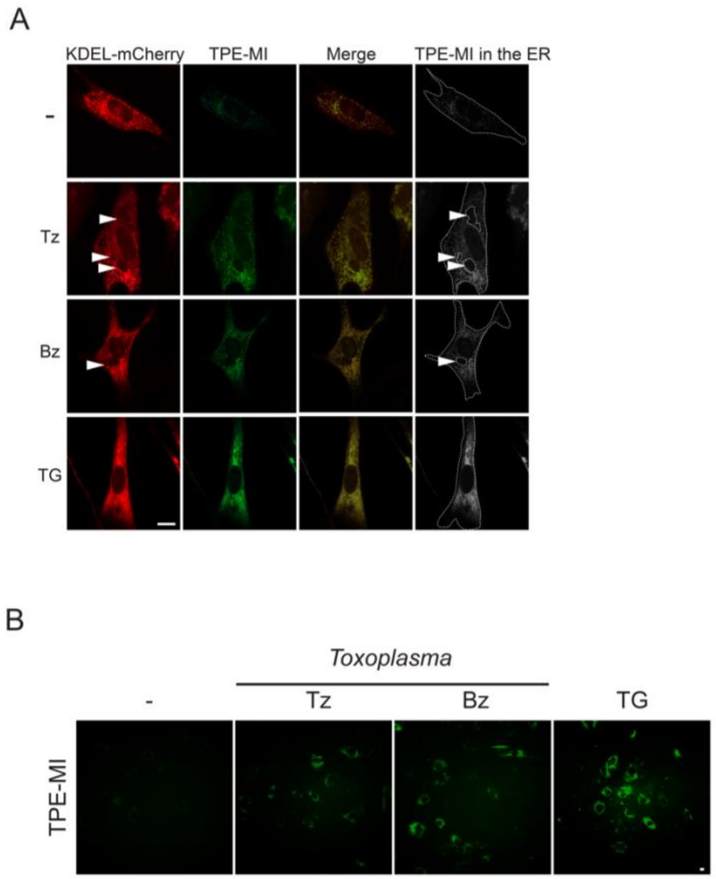

Supplementary Figure 1. Live cell image of cells treated with TPE-MI. Cells were incubated with TPE-MI for 10 min. (A) The intensity of TPE-MI was determined by live cell imaging and quantified using ImageJ. The positive control consisted of uninfected cells treated with 1μM Thapsigargin (TG) for 6h, an ER stress inducer. Scale bar = 5 μm. The arrow indicates the parasite vacuole. These are representative images from Figure 1B. At least 50 cells were imaged per condition for each of three independent experiments. (B) 20x magnitude of TPE-MI treated cells. Scale bar = 5 μm.

Supplementary Figure 2

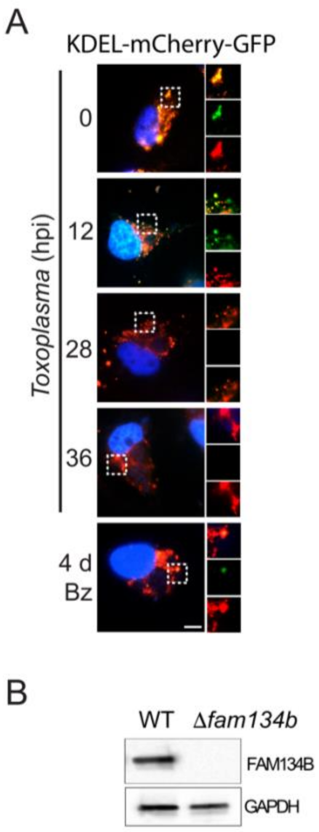

Supplementary Figure 2. (A) ER-phagy were infected with *T. gondii* at different time points. as indicated, and the GFP and mCherry foci were quantified by cell imaging. (B) The CRISPR/Cas9-engineered depletion of FAM134B, designated *Δfam134b,* was assayed by immunoblotting using FAM134B antibody compared to WT cells (GAPDH was included as a loading control).

Supplementary Figure 3

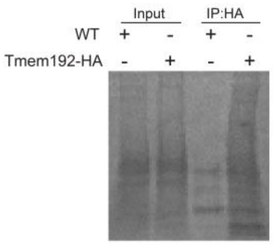

Supplementary Figure 3. Ponceau's staining of the membrane referred to in Figure 4D.

Supplementary Figure 4

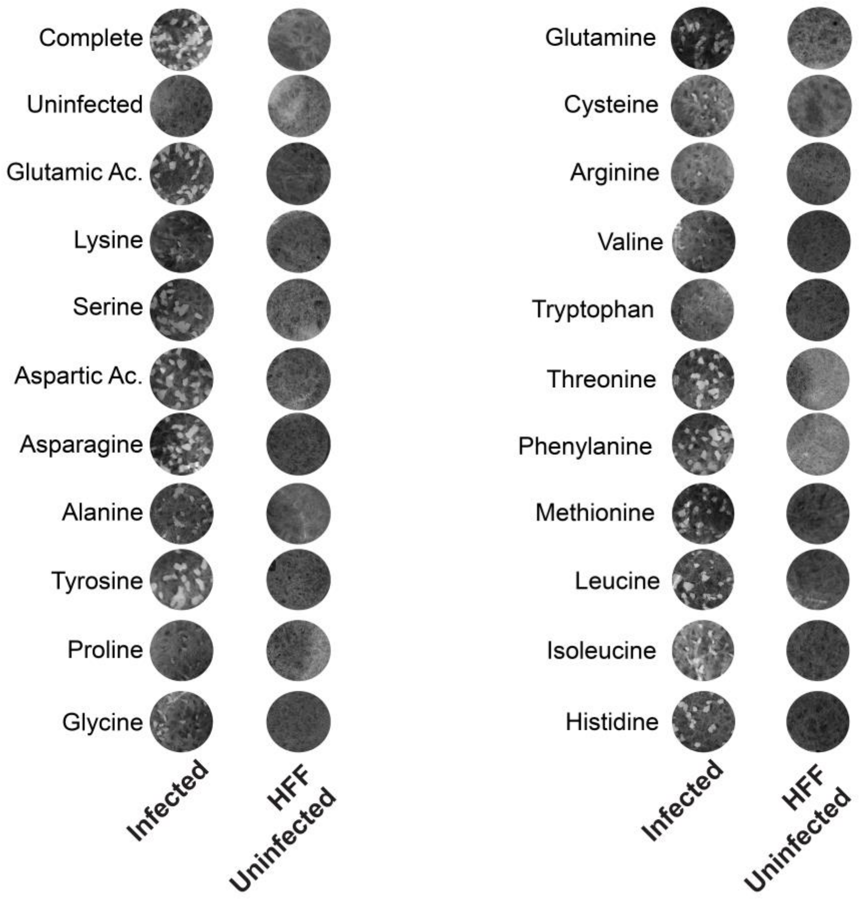

Supplementary Figure 4. Representative images of plaque assay of infected and uninfected cultivated in individual amino acid depleted media (referred to Figure 4F).
